# REGene: a literature-based knowledgebase of animal regeneration that bridge tissue regeneration and cancer

**DOI:** 10.1038/srep23167

**Published:** 2016-03-15

**Authors:** Min Zhao, Bronwyn Rotgans, Tianfang Wang, S. F. Cummins

**Affiliations:** 1 School of Engineering, Faculty of Science, Health, Education and Engineering, University of the Sunshine Coast, Maroochydore DC, Queensland, 4558, Australia

## Abstract

Regeneration is a common phenomenon across multiple animal phyla. Regeneration-related genes (REGs) are critical for fundamental cellular processes such as proliferation and differentiation. Identification of REGs and elucidating their functions may help to further develop effective treatment strategies in regenerative medicine. So far, REGs have been largely identified by small-scale experimental studies and a comprehensive characterization of the diverse biological processes regulated by REGs is lacking. Therefore, there is an ever-growing need to integrate REGs at the genomics, epigenetics, and transcriptome level to provide a reference list of REGs for regeneration and regenerative medicine research. Towards achieving this, we developed the first literature-based database called REGene (REgeneration Gene database). In the current release, REGene contains 948 human (929 protein-coding and 19 non-coding genes) and 8445 homologous genes curated from gene ontology and extensive literature examination. Additionally, the REGene database provides detailed annotations for each REG, including: gene expression, methylation sites, upstream transcription factors, and protein-protein interactions. An analysis of the collected REGs reveals strong links to a variety of cancers in terms of genetic mutation, protein domains, and cellular pathways. We have prepared a web interface to share these regeneration genes, supported by refined browsing and searching functions at http://REGene.bioinfo-minzhao.org/.

Animal regeneration refers to the regeneration of damaged or diseased body parts to completely restore function^1^,^2^. It involves stem cells that have the capacity to differentiate and mature into a variety of cell types depending on the potency of the stem cell and the organism. In fact, the ability to regenerate is vastly different across the animal kingdom. In metazoans, animal groups like: hydra, planaria, starfish and several worms can regenerate their entire body from a small body fragment^3^, whereas birds, nematodes and leeches have lost all capacity for self-renewal^2^.

The majority of human tissues and organs possess limited self-renewal and true-regeneration abilities, which is not to be confused with compensatory growth, the mechanism by which tissues such as the liver recover from trauma. Regenerative medicine is an area that promises to repair damage following traumatic injury or disease, by direct stimulation of a wound-site, or by introduction of exogenous, man-made tissue^4^. Multiple therapeutic strategies are being explored including: small molecules, gene delivery, and stem cells. Recent advances in tissue engineering provide more practical approaches to achieving regeneration; tissue engineering can enhance the regenerative cascade and stimulate production of the body’s own complex tissues by replacing lost or damaged material^5^. However, progress with transplantations has been hampered due to the complexity of the interactions and regulatory systems involved, as well as the sheer diversity of tissues and organs these cells differentiate into.

The molecular mechanisms of regeneration are well studied in several model organisms. For example, the SemdGD and Planform databases were developed to browse the genomes of regenerative free-living species, including *Schmidtea mediterranea*; a freshwater planarian with a capacity to regenerate from small body fragments into a complete body^6^,^7^. Additionally, numerous studies have focused on limb regeneration, which have been systematically combined into the Limbform resource^8^. However, these studies are focused specifically on a limited number of species or only limb regeneration, so the broader view regarding multi-species organ/tissue regeneration is still lacking. Moreover, the differences and similarities of different regenerative processes is unclear. To elucidate the commonalities, the data must be mined systematically for all kinds of regeneration and integrated into one resource to provide us with the essential knowledge to eventually understand, manipulate and control regenerative properties. The majority of regeneration studies to date have not focused beyond the gene level. Although, with the development of affordable high-throughput sequencing technology, a few studies have characterized the change in gene expression during limb regeneration in salamanders^9^, fin, heart and retinal regeneration in zebrafish^10^, and fin regeneration in medaka^11^. Furthermore, numerous microRNAs have now been identified as regeneration genes^12^,^13^,^14^, which further adds to the complexity of the regenerative cellular signaling map. Importantly, these studies lack cross-species data integration, and thus fail to provide the whole picture of regenerative cellular processes. In addition, the relationship of regenerative process and other common diseases such as cancer are unexplored systematically, although there are some clues documented^15^.

In this study, we curated genes with identified links to regeneration, from an array of tissue types and species listed in 1293 PubMed abstracts. Additionally, well annotated regeneration genes from the gene ontology (GOA) database^16^ were integrated to produce a total of 948 human regeneration-related human genes, and 8445 homologs from another 11 species was obtained. Moreover, we provide high quality annotations detailing biological pathways, gene expression, regulation, and interaction, to aid regeneration researchers in obtaining a rapid understanding of the known molecular mechanism for regeneration in various tissue/organs. This data resource also makes it feasible to prioritize genes by their regeneration-associated importance and to identify both the common and unique cellular events involved in different regenerative processes.

## Results and Discussion

### Data integration and literature search

**Figure 1:**
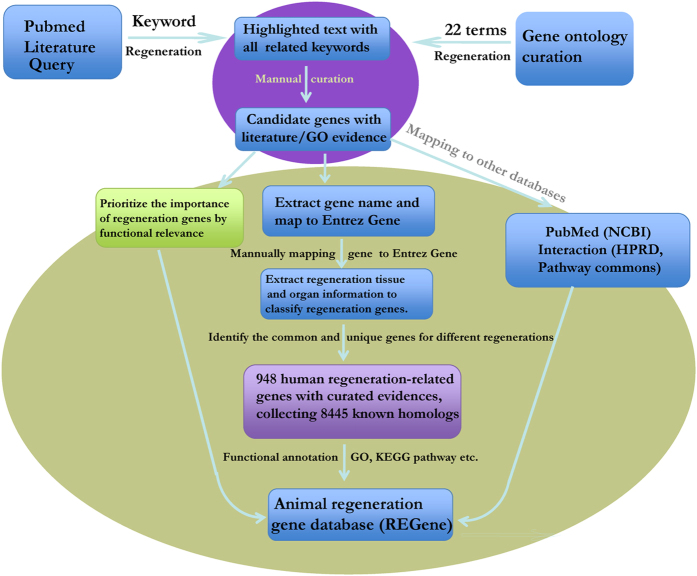
Pipeline for collection and annotation of regeneration-related genes.

The primary aim for the REGene database was to collect and maintain a high quality animal regeneration gene resource, which serves as a comprehensive, classified, and accurately annotated regeneration gene knowledgebase. The database provides extensive cross-references and querying functionality. It is in the public domain and freely accessible to support the animal regeneration and regenerative medicine research community in the design of systematic regeneration and regenerative medicine studies. In order to provide a comprehensive resource, we collected known regeneration genes from the gene ontology annotation database (GOA)^16^ and GeneRif literature database^17^ (Fig. 1). To retrieve a comprehensive list of annotated genes from GOA, we curated 20 GO terms related to regeneration and extracted 549 genes from the GOA database associated with regeneration GOs (see methods for GO terms). Due to the pace of research in this field and the volume of data generated, GOA annotation does not always provide the most up-to-date literature to support regeneration gene roles, data curation is, by it’s nature, always a step behind regenerative biology research.

To provide a detailed and precise regeneration gene resource with literature evidence, we performed an extensive literature query of GeneRif (Gene Reference Into Function) database (17/12/14) using the keyword “regeneration”, resulting in a return of 2245 PubMed abstracts. GeneRIF is a collection of short gene function descriptions for entries in the Entrez Gene database^17^. To ensure the precision of collected regeneration information, much care was taken regarding species information and the regenerative organ/tissue. For example, in the sentence “*ACF regulates liver regeneration following partial hepatectomy at least in part by controlling the stability of IL-6 mRNA*”^18^ the gene *ACF* was listed as a synonym for mouse *A1cf* in the current Entrez gene database. Following careful manual inspection, the list was refined to 1417 Entrez genes from various species, obtained from 1293 PubMed abstracts. To provide a more comprehensive overview, we mapped all the 1417 genes to 936 homologous groups using the NCBI HomoloGene database, as has been implemented in previous analysis^19^,^20^,^21^,^22^. By assimilating the regeneration-related genes from GOA, we consolidated our list for further annotation and database construction to 948 human genes including 929 protein-coding and 19 non-coding genes (Table S1). Using these human genes, we were able to retrieve 8445 homologs from 17 experimental model organisms using the HomoGene database.

### Representative entry in REGene

**Figure 2:**
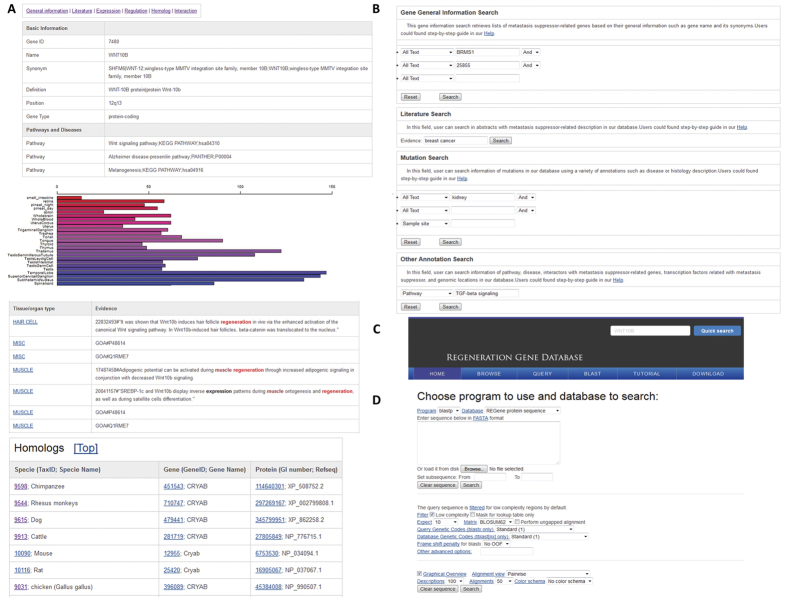
Web interface of REGene. (**A**) The basic information in each regeneration-related gene page. The expression values in the bar represent the relative expression scores from BioGPS database. (**B**) Query interface for text search; (**C**) Quick search button for gene symbol-based search. (**D**) BLAST search interface for comparing query against all sequences in REGene.

To provide data access for the regeneration community, we constructed a web-based platform, REGene, to store all the information for REGs. As shown in Fig. 2, a typical REGene gene entry contains six categories of information, accessible by clicking the labels: “General information,” “literature,” “Expression,” “Regulation,” “Homolog,” and “Interaction” displayed on the top of the page. The basic information, including: gene name, pathway, disease-association, nucleotide sequence, and protein sequence, can be found in a tabular view in the “General information” page (Fig. 2A). Highlighted summaries of supporting literature and gene ontology annotation sources are provided in the “literature” page (Fig. 2A). While on the “Expression” page, gene expressions from 84 normal tissues and 184 tumor samples are piled using a bar plot with the sample name and normalized expression scores (Fig. 2A), which is useful in exploring the tissue specificity of each regeneration gene among normal and tumor samples. Take the gene *WNT10B* as an example: the expression bar view indicates that it is expressed relatively high in certain brain regions: the temporal lobe and the superior cervical ganglion (Figure S1). The “homolog” page allows the user to map human genes to 17 model species, including a filamentous fungus (*Ashbya gossypii*), Baker’s yeast (*Saccharomyces cerevisiae*), Cattle, Chicken, Chimpanzee, Dog, Fission yeast, Frog, Fruit fly, Milk yeast, Mosquitos, Mouse, Neurospora, Rat, Rhesus monkeys, Worm, and Zebrafish. Additionally, the sequences in the page allow the user to easily retrieve the sequences for phylogenetic relationship analysis (Figure S2). The “Regulation” page is designed to classify regulatory information, including: interactions with transcription factors, abundance of post-translational modification information, and methylation features for each REGs. For those interested in systems biology, the interaction partners of each REGs are presented in the “Interaction” page to illustrate different interaction categories including: physical interactions from high-throughput experiments, as well as metabolic and signaling interactions from known pathway databases^23^.

In order to accommodate a broad range of user queries against our REGene data, we developed six powerful query platforms: pathway and disease information, genomic location, literature evidence, and gene expression range in human samples, and homology information (Fig. 2B). Notably, a quick text search for the GeneID, gene symbol, and gene alias exists on the top right of each page, to allow the user to retrieve any desired information from the database quickly (Fig. 2C). Users can run a sequence similarity search (BLAST) against the nucleotide and protein sequences in REGene (Fig. 2D), or explore other features of the data including: the organ/tissue type, significantly enriched pathway, related disease, reported linkage region, and chromosome number. For each related KEGG pathway, the marked chart is provided to highlight all the known regeneration-related genes (Figure S3). Finally, for the purpose of offline data usage, we provide a downloadable plain text format gene list corresponding to all the organ/tissue types for all 948 regeneration related genes collected.

### Functional analysis of human REGs revealed an enrichment of cell proliferation and developmental processes

To explore the biological processes associated with our collected genes, gene-set enrichment analysis was adopted, characterizing whether the 929 human protein-coding REGs had any significant annotations as compared to all human protein-coding genes. A strict cutoff was implemented (corrected P-value less than 0.01 and the annotated genes more than 30% of all 929 REGs), we were able to identify 30 gene ontology (GO) terms (Table 1), and 17 statistically significant enriched phenotypes (Table S2). The enriched GO terms identified are chiefly related to cell proliferation and development, specific examples include: regulation of developmental processes, tissue development, and regulation of cell proliferation (Table 1). Interestingly, the enriched GOs also include cellular processes in response to wounding, oxygen-containing compounds, and endogenous stimuli. This finding aligns with studies in zebrafish that have demonstrated that low oxygen (hypoxia) can adversely affect heart regeneration^24^. The other GO clusters are associated with cell apoptosis, metabolism and locomotion. For the 17 enriched phenotypes, the majority relate to abnormal organ morphology and physiology, such as: abnormal cardiovascular system, and immune system morphology/physiology. Moreover, at least 437 REGs represent essential genes related to “prenatal lethality” or “lethality during fetal growth through weaning” in mouse models. These huge numbers of essential genes in human REGs also highlight their critical roles in organism development.

### Enriched REGs encode proteins involved in cancer-related processes and contain domains highly affiliated with cancer

Further gene set enrichment analyses; for diseases, pathways, and protein domains, revealed that human REGs are enriched with cancer-related signaling pathways and domains such as PI3K-Akt signaling pathway, and EGF domains (Tables S3 and S4). To explore the role of REGs in specific cancers, all REGs were mapped to a KEGG colorectal cancer and pancreatic cancer pathway; as shown in Figure S3, over 90% of genes associated with colorectal and pancreatic cancer pathways are REGs. The specific connections between REGs and a broad-spectrum of human adult cancers (Table S3) may be able to provide a better understanding of common mechanisms utilized by both processes. To date, few studies in the scientific have linked tissue regeneration with cancer^15^,^25^,^26^,^27^,^28^. Importantly, the enrichment analysis of REGs does not quantitatively measure the degree of commonality between the molecular mechanisms that underpin regeneration and cancers, rather it implies that the relevant signaling pathways of the two are very similar. This link is not limited to providing insight into the cellular process, but also suggests a cancer-like regulation of regenerating tissue. For instance, 12 intestine REGs are enriched in colorectal cancer gene sets (corrected P-value = 0.00042). By the same token, systematic comparison of regeneration, using the REGene database with specific diseases may provide a more comprehensive picture for the underlying molecular mechanisms of the two processes, both in terms of the particular tissue inspected, and more holistically. For example, 22 heart REGs are associated with coronary artery disease (corrected P-value = 0.0086), this suggests certain signaling components/pathways are shared by these two vastly different processes.

A key finding in our analysis was identifying 54 REGs that contain epidermal growth factor (EGF)-related domains. These over-represented EGF domains are EGF-1, EGF-2, EGF-3 and EGF-like domains. EGF proteins have profound roles in various regenerative processes, including liver regeneration^29^ and regulation of hematopoietic regeneration after radiation-damage^30^. At the same time, the EGF-related family has been implicated in carcinoma cell growth and survival, through multiple ligands to induce cell transformation^31^. Shared EGF-related proteins and relevant downstream pathways further solidify the link between regenerative processes and complex diseases like cancer. Consequently, further research regarding EGF-related REGs has the potential to not only deepen our insight in the regenerative biology field, but may direct the development of potential anti-cancer therapeutics targeting EGF pathways.

### Common REGs across multiple regenerative tissue types are shared with cancers

**Figure 3:**
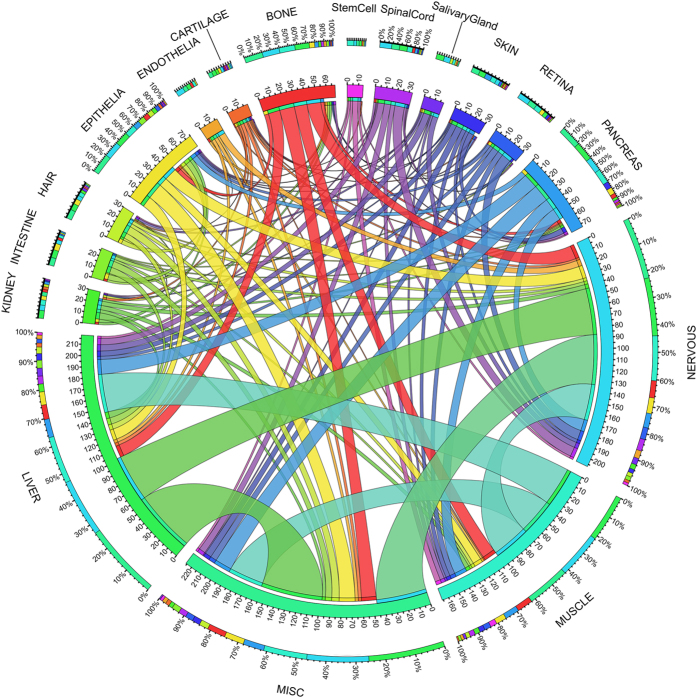
Shared regeneration-related genes across multiple regenerative processes. The length of circularly arranged segments is proportional to the total genes in each regenerative process group. The ribbons connecting different segments represent the number of shared genes between regenerative process groups. The outer ring is stacked bar plots that represent relative contribution of other regenerative process group to the regenerative process group totals. Ribbons connecting different segments represent the number of shared genes between regenerative tissues. The 17 regenerative tissue/organs are bone, cartilage, endothelia, epithelia, hair cell, intestine, kidney, liver, muscle, nerves, pancreas, retina, salivary gland, skin, spinal cord, stem cells and miscellaneous (short with MISC).

Information derived from the existing regeneration literature facilitated gene annotation for all REGene entries with a specific tissue/organ type. Tissue/organ types were collected into 17 major groups of regenerative tissue: bone, cartilage, endothelia, epithelia, hair cell, intestine, kidney, liver, muscle, nervous system, pancreas, retina, salivary gland, skin, spinal cord, stem cells, and miscellaneous. The majority of human REGs were identified from nerve (284 genes, 29.95% of total 948 REGs), liver (246 genes, 25.95%) and muscle (197 genes, 20.78%) tissues. The relationships of common genes that were identified in multiple regenerative tissue/organs were plotted (Fig. 3). This suggests that the molecular machinery adopted by regenerative processes in different tissues possess uniform components, a feature that could logically be attributed to either evolutionary expediency, or functional importance. In total, 149 human REGs were involved in regeneration by 2 tissue types. In addition, 85 human REGs were determined to be shared by 3 or more regenerative tissues. This large number adds further weight to the conjecture that the regenerative process in multiple tissue types share molecular mechanisms. In addition to this, further functional enrichment analysis on these 85 REGs not only confirmed their roles in regeneration (P-value = 6.75e-12, Table S5), but also linked the REGs to a multitude of cancer types, including: bladder cancer, breast cancer, colorectal cancer, endometrial cancer, kidney cancer, oral cancer, pancreatic cancer, prostate cancer, and stomach cancer (all corrected P-value are less than 0.05, Table S5). In conclusion, the large overlap observed for common REGs with cancer pathways points to shared molecular mechanisms for tissue regeneration and cancer progression.

### Prioritization of key genes in animal regeneration reveals abundant mutations across multiple cancer types

To systematically evaluate the importance of regeneration-related genes, we conducted a gene ranking analysis, using ToppGene (see methods) with a training set of 19 reliable genes supported at least 10 times within the literature. The resultant top ten ranked genes consisted of: APC, ERBB2, MTPN, PTEN, CDH1, CDKN2A, MCAM, FGL1, MIR204, MIRLET7A1 (Table S6). Not surprisingly, the majority of these genes are components of pathways regulating cell proliferation and tumorigenesis such as the cell cycle control and DNA damage pathway.

**Figure 4:**
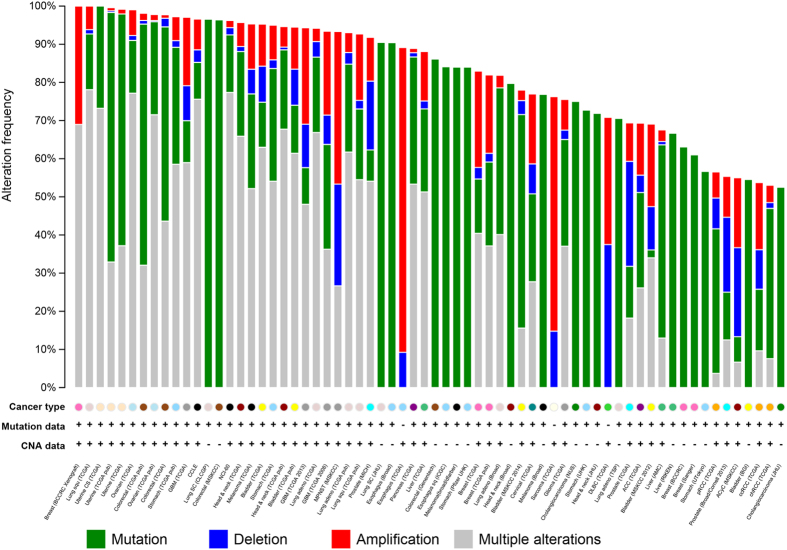
The mutational landscape for the top 100 ranked regeneration-related genes in multiple cancers. The CAN represent copy number alteration. The presentation of any mutations in a cancer types are indicted with “+”. The lacking of any specific mutations are “−”. Same cancer types are marked as the same color.

Although these REGs are over-represented in a number of cancers, the systematic examination of genetic variants in multiple cancers requires further investigation. Such mutation patterns could vastly augment comparisons of REGs with their anatomically-corresponding cancers. With comprehensive cancer genomics datasets available via The Cancer Genome Atlas (TCGA) project, there exists an unprecedented opportunity for exploring the global genetic mutation of REGs in multiple cancer types. As shown in Fig. 4, the top 100 ranked REGs (comprised of 81 top ranked genes and the 19 genes used as the ToppGene training set) have an overwhelming number of mutations in cancers (Table S7); these 100 genes are mutated in over 90% of patients across 30 different cancer types. A most striking case exemplifying this can be observed analyzing the lung squamous cell carcinoma cohort comprised of 178 patients, who all presented with mutations in the top 100 REGs. In like fashion, the top 100 REGs are mutated in (99.60%) of a 239-strong cohort of Uterine Corpus Endometrioid Carcinoma patients; over half of which were single nucleotide mutations. The very same pattern can be seen in a host of other cancers: colorectal cancer from the TCGA dataset (98.10% in 208 individuals) and ovarian cancer (99% in 308 individuals). As summarized in Table S7, the top 100 REGs have mutations in over 50% of patients across 67 major cancer types. This result strongly suggest REGs may have important roles for cancer progression, roles that are shared in various cancers, further comparison between the regenerative process of specific tissues and corresponding cancer types may provide a thorough intimation of the nature of these caner-connections.

Mutation frequency on the protein domain level was further explored within the 19 REGs implemented earlier as a training set for ToppGene (AKT1, BDNF, BMP2, CTNNB1, CXCL12, EGFR, FGF2, GAP43, HGF, IGF1, IL6, MET, RTN4, RTN4R, SOCS3, STAT3, TGFB1, TP53, VEGFA) supported at least 10 times within the scientific literature. As shown in Table S8 and Figure S4: AKT1 has variations in 108 samples from 16 adult cancers (bladder, breast, cervical, colorectal, glioblastoma, head and neck, liver, lung adenocarcinoma, lung squamous cell carcinoma, melanoma, pancreas, papillary renal cell carcinoma, prostate, stomach, thyroid, uterine cancer). In total, the 19 REGs possess 8221 mutation events in multiple cancer types, mainly concentrated in regions encoding protein functional domains. To put it succinctly, a great many well-known cancer genes, such as TP53, EGFR and AKT1, have prominent roles in the regeneration processes; this striking overlap of genes and pathways is indicative of an, as yet, unexplored connection between cancer and regeneration.

### Reconstructed REG protein-protein interaction network exhibits a highly modular structure

**Figure 5:**
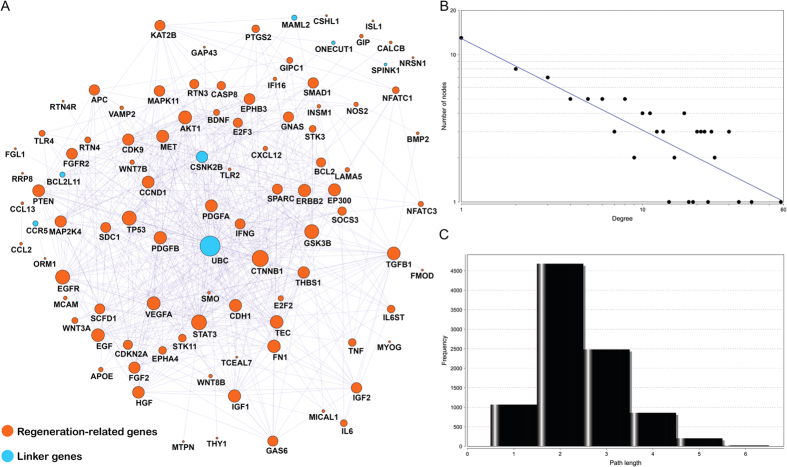
Reconstructed regenerative cellular map using pathway-based protein-protein interaction data. (**A**) The 90 genes in orange are genes in our REGene; the remaining 7 genes in blue are linker genes that connect the 90 genes; the size of the node represents the number of connections in the network; (**B**) Degree distribution; (**C**) Short path length frequency.

To develop a thorough picture of the regenerative processes and construct the most comprehensive cellular map of regeneration, the connections among top ranked REGs, as recorded in reliable public data sources, were explored. The top ranked 100 REGs were incorporated into an interactome from the Pathway Commons database, which combines all prevailing pathway databases to provide functional gene-gene interaction pairs^23^. The extracted sub-network of REGs contains 97 genes and 534 gene-gene interactions (Fig. 5A). It is worth noting that all interactions are based on current evidence from known biological pathways with biological meaning, not physical interactions from high-throughput experiments (Fig. 5A). Of the 97 nodes, 90 are among our top 100 ranked REGs; the remaining 7 are linker genes that connect REGs facilitating their cellular function. The vast majority of top ranked REGs are linked to each other in such a way as to form highly modular structures. This serves to further verify our earlier deductions, and to reveals that REGs are highly connected to each other, assuming a high-density modular structure.

Further topological analysis of the REGs network reveals a high degree of interconnectivity amongst each other. Only 14 nodes were limited to one connection (Fig. 5B), this implies that the majority of nodes are capable of communicating with each rapidly and with great ease across short paths. The degrees of all nodes in our regeneration map follow a power law distribution *P(k)~*^−*b*^, where *P(k)* represents the probability that a gene has links with *k* other genes while *b* represents an exponent with an estimated value of 0.622. The resultant map of REG networks is quite different from other human PPI (Protein-protein interaction) networks where most nodes are sparsely connected, with an exponent *b* of 2.9^32^. This topological feature indicates a high degree of connectivity, with the shortest path length distribution for the network being a relatively smaller number: 2 and 3, meaning ~76.9% of node communication can be reached in only two or three steps (Fig. 5C). With high modularity, the hub nodes in this network may have prominent roles, these nodes act as common connections to mediate rapid and efficient information transfer. In total, there are 6 genes with ≥30 connections: UBC (58 connections), CTNNB1 (40), STAT3 (34), TP53 (30), GSK3B (30), and EGFR (30). With the exception of UBC, all these hub genes are from our literature-based gene set. To be concise: Network analysis of REGs identified novel linker gene hubs that are undoubtedly crucial to regenerative processes in addition to revealing a highly modular structure to the network of all analyzed regenerative genes.

### Conclusion and future plan

REGene is the first literature-based gene resource dedicated to furthering animal research by integrating multi-dimensional bioinformatics data consisting of: gene expression, regulation, homology, and interactions. It should prove a valuable tool to probe the molecular mechanisms underpinning animal regeneration and thus expedite the development of regenerative medicine therapies. The REGene database is in the public domain and freely accessible at http://regene.bioinfo-minzhao.org/.

The high heterogeneity of cellular processes presents an enormous challenge towards understanding animal regeneration. Classical approaches for the identification of candidate genes that relate to specific regenerative phenotypes have been conducted, however, these studies seldom incorporate multiple species comparisons. Following on from the REGene, we plan to integrate other homologous genes from other species with regenerative capacities, including salamanders, axolotls, and from other species of hydra and planarian. Also starfish, where there has been accumulating gene resources becoming available. This information will further enable a comparative systems biology approach to summarize the commonality and uniqueness of animal regeneration, removing bias resulting from any single species study or technology platform. For cancer-related study, it will also be interesting to compare the REGs with other cancer-related processes^33^,^34^,^35^ or genes on specific cancer types^36^,^37^,^38^. We will continue to maintain and update the REGene database, as new research references appear, particularly data from large-scale genomics studies such as ChIP-seq. Since our study indicated that many regeneration-related genes are involved in cancer progression, we also plan to integrate high-throughput cancer genomics data.

## Methods

### Data collection

To collect the regeneration-related genes, the gene ontology annotation database was downloaded on Dec 8^th^, 2014^16^. 20 gene ontology (GO) terms related to regenerative processes were collected as follows: axon extension involved in regeneration (GO:0048677), axon regeneration (GO:0031103), cardiac muscle tissue regeneration (GO:0061026), collateral sprouting of injured axon (GO:0048674), dendrite regeneration (GO:0031104), fin regeneration (GO:0031101), formation of growth cone in injured axon (GO:0048689), liver regeneration (GO:0097421), MAPK cascade involved in axon regeneration (GO:1903616), myoblast differentiation involved in skeletal muscle regeneration (GO:0014835), myoblast migration involved in skeletal muscle regeneration (GO:0014839), myotube differentiation involved in skeletal muscle regeneration (GO:0014908), neuron projection regeneration (GO:0031102), organ regeneration (GO:0031100), peripheral nervous system axon regeneration (GO:0014012), regeneration (GO:0031099), sensory epithelium regeneration (GO:0070654), skeletal muscle satellite cell maintenance involved in skeletal muscle regeneration (GO:0014834), skeletal muscle tissue regeneration (GO:0043403), tissue regeneration (GO:0042246).

To further curate matched literature, 2245 PubMed abstracts associated with regeneration were downloaded for manual review. Curation of regeneration genes from literature included three major steps: (1) grouping all 2245 PubMed abstracts by topic, using the “Related Articles” function in Entrez; (2) extracting descriptions of regeneration genes from grouped abstracts; (3) manually collecting gene names from the descriptions of the regeneration genes and mapping the gene names to Entrez gene IDs. These three steps allowed us to quickly and easily evaluate if, and how, the curated abstracts were related to regeneration genes while allowing for cross validation between multiple literature sources. Here, Entrez gene IDs for regeneration genes served as the REGene database’s crosslink between the same genes from different public databases.

### Database interface

The REGene database is written in Perl CGI and JavaScript. The database manage system is MySQL which stores the relationship data model. The website is served with Apache on a server running Red Hat 4.4.7-11. The dynamic coding is mainly implemented using two primary components: web applications for browsing and searching, and CSS that control the general page style of visualization.

### Biological functional annotations and database construction

To better understand the function of the regeneration genes collected into the REGene database, associated functional information for each gene was collected. Representative annotations in the REGene database are summarized in Table 1. Basic gene information is included, such as gene names from the Entrez gene database^39^, crosslinks to the rate-limiting enzyme RLEdb^40^, text mining server iHOP^41^. For functional annotations, the pathways involving the genes were retrieved from BioCyc^42^, KEGG Pathway^43^, PID Curated^44^, PathLocdb^45^, PANTHER^46^, and PID Reactome^47^,^48^; possible association with diseases were also extracted from KEGG Disease^49^, Fundo^50^,^51^, GAD^52^, NHGIR^53^, and OMIM^54^ using the functional annotation server KOBAS^55^,^56^. Additionally, possible post-translational modifications and transcription factor regulation information was collected from dbPTM^57^ and the TRANSFAC database^58^, respectively. Digital gene expression information for 184 tumor samples and 84 normal tissues were integrated from BioGPS^59^; While Information about methylation sites, and protein-protein interactions were integrated from DiseaseMeth^60^, and Pathway Commons^23^ databases, respectively.

### Gene ranking using ToppGene and cancer mutational pattern analysis

Hundreds of genes were collected that originated from various organ/tissue types, although the common REGs were still unclear. All REGs were scattered in individual studies, which often focus on verifying highly specific tissue/organ regeneration. Thus, data integration and evaluation across all the regenerative types may help to highlight some important common REGs and their global involvement in regenerative processes. To this end, the ToppGene gene ranking tool^61^ was used to prioritize all the 948 genes in the REGene database. Essentially, the ToppGene tool extracts features based on a training gene list by using a multiple dimensional dataset, including biological annotations, gene expression, sequence features, protein-protein interaction, and literature evidence. In this analysis, the training set was comprised of 19 well-known regeneration-related genes (*AKT1, BDNF, BMP2, CTNNB1, CXCL12, EGFR, FGF2, GAP43, HGF, IGF1, IL6, MET, RTN4, RTN4R, SOCS3, STAT3, TGFB1, TP53, VEGFA*) that were supported within scientific literature by a minimum of 10 studies. The resultant gene-prioritizing model input the remaining 921 genes and integrates all the outputs from the training models to form a global ranking for all the candidate REGs.

Based on the curated gene information, all organ/tissues types were collected into 17 major groups according anatomic and biological functions. The overlapping cancer genes across cancer types were visualized using Circos^62^. While mutational landscape in multiple cancer types for the top 100 ranked REGs were generated using the cBio portal^63^.

### Functional enrichment analysis

Throughout this research, the representative pathways from KEGG and Reactome for each gene set were identified by KOBAS^41^. In this pathway analyses, all human protein-coding genes were set as background in order to calculate statistical significance. In addition, the Benjamini-Hochberg multiple testing corrected P-values for enriched pathways were adopted based on hypergeometric test by using KOBAS. Finally, enriched human pathways with corrected P-values less than 0.01 were identified as over-representative pathways for each gene set.

### Reconstructing a protein-protein interaction network related to REGs

To explore the relevant biological mechanisms related to REGs, all protein-protein interactions associated with the 948 REGs were extracted. To this end, we used a non-redundant human interactome from the PathCommons database^23^, containing 3629 proteins and 36,034 protein-protein interactions. It is of note that the collected protein-protein interactions are from pathway databases (HumanCyc^42^, Reactome^36^,^37^, and KEGG pathway^43^), which have biological meaning, rather than physical interaction. Thus, the final interactome is comprised of pathway-based interactions. To extract a sub-network related to the top 100 ranked REGs, we used the similar approach implemented in our previous study^64^. In this algorithm, all the 100 REGs were mapped to the human pathway-based interactome, which was used to produce a sub-network with as many input genes connected by their shortest path as possible.

Generally speaking, biological networks are extremely complex, but often follow a few simple rules that may relate to their function^65^. Essentially, the topological properties of networks can yield clues that reveal elements of their function. To explore the REGs interactome, the NetworkAnalyzer plugin in Cytoscape 2.8 was used to calculate the topological properties of the REG network (Fig. 5B,C)^66^, the amount of connections at each node was represented as the degrees in the network^65^. Finally, path distribution was calculated to reveal the shortest route for any one node to reach another^65^. The final network visualization was generated using Cytoscape^66^.

## Additional Information

**How to cite this article**: Zhao, M. *et al.* REGene: a literature-based knowledgebase of animal regeneration that bridges tissue regeneration and cancer. *Sci. Rep.* **6**, 23167; doi: 10.1038/srep23167 (2016).

## Supplementary Material

Supplementary Information

Supplementary Dataset 1

Supplementary Dataset 2

Supplementary Dataset 3

Supplementary Dataset 4

Supplementary Dataset 5

Supplementary Dataset 6

Supplementary Dataset 7

Supplementary Dataset 8

## Figures and Tables

**Table 1:** Summary of statistically significant enriched gene ontology annotations of regeneration-related genes.

Gene ontology	Adjusted P-values^*^
Regulation of developmental process	6.59E-169
Cell proliferation	9.02E-168
Tissue development	1.56E-163
Regulation of multicellular organismal development	2.84E-154
Regulation of cell proliferation	9.99E-153
Response to wounding	1.07E-150
Cellular response to organic substance	2.71E-137
Response to oxygen-containing compound	8.51E-136
Regulation of cell differentiation	6.12E-135
Cell development	1.43E-134
Response to endogenous stimulus	4.66E-134
Regulation of cell death	7.46E-124
Regulation of programmed cell death	1.63E-117
Locomotion	3.17E-117
Positive regulation of response to stimulus	3.99E-117
Regulation of apoptotic process	8.99E-115
Positive regulation of cell communication	3.05E-114
Positive regulation of signaling	3.68E-114
Programmed cell death	4.39E-114
Movement of cell or subcellular component	1.20E-112
Apoptotic process	2.61E-111
Neurogenesis	3.02E-109
Positive regulation of biosynthetic process	2.56E-106
Positive regulation of cellular biosynthetic process	6.38E-104
Receptor binding	8.05E-103
Generation of neurons	6.75E-102
Regulation of cellular component organization	9.89E-96
Positive regulation of macromolecule biosynthetic process	2.36E-94
Positive regulation of nitrogen compound metabolic process	7.89E-94
Positive regulation of nucleobase-containing compound metabolic process	6.61E-92
Phosphorylation	2.52E-83
Transcription from RNA polymerase II promoter	2.11E-69
Negative regulation of metabolic process	9.26E-69
Regulation of phosphate metabolic process	5.76E-65
Regulation of phosphorus metabolic process	2.68E-64

Note: ^*^Adjusted *P*-values: the *P*-values of the hypergeometric test were corrected by Benjamini-Hochberg multiple testing correction.

## References

[b1] Brockes J. P. & Kumar A. Comparative aspects of animal regeneration. Annu Rev Cell Dev Biol 24, 525–549 (2008). 18598212 10.1146/annurev.cellbio.24.110707.175336

[b2] Li Q., Yang H. & Zhong T. P. Regeneration across Metazoan Phylogeny: Lessons from Model Organisms. J Genet Genomics 42, 57–70 (2015). 25697100 10.1016/j.jgg.2014.12.002

[b3] Sanchez Alvarado A. Regeneration in the metazoans: why does it happen? Bioessays 22, 578–590 (2000). 10842312 10.1002/(SICI)1521-1878(200006)22:6<578::AID-BIES11>3.0.CO;2-#

[b4] Hosseinkhani M. *et al.* Tissue engineered scaffolds in regenerative medicine. World J Plast Surg 3, 3–7 (2014). 25489516 PMC4236978

[b5] Feinberg A. W. Engineered tissue grafts: opportunities and challenges in regenerative medicine. Wiley Interdiscip Rev Syst Biol Med 4, 207–220 (2012). 22012681 10.1002/wsbm.164

[b6] Robb S. M. *et al.* SmedGD 2.0: The Schmidtea mediterranea genome database. Genesis 53, 535–546 (2015). 26138588 10.1002/dvg.22872 PMC4867232

[b7] Lobo D., Malone T. J. & Levin M. Planform: an application and database of graph-encoded planarian regenerative experiments. Bioinformatics 29, 1098–1100 (2013). 23426257 10.1093/bioinformatics/btt088 PMC3624808

[b8] Lobo D. *et al.* Limbform: a functional ontology-based database of limb regeneration experiments. Bioinformatics 30, 3598–3600 (2014). 25170026 10.1093/bioinformatics/btu582 PMC4253831

[b9] Monaghan J. R. *et al.* Microarray and cDNA sequence analysis of transcription during nerve-dependent limb regeneration. BMC Biol 7, 1 (2009). 19144100 10.1186/1741-7007-7-1 PMC2630914

[b10] Cameron D. A., Gentile K. L., Middleton F. A. & Yurco P. Gene expression profiles of intact and regenerating zebrafish retina. Mol Vis 11, 775–791 (2005). 16205622

[b11] Katogi R. *et al.* Large-scale analysis of the genes involved in fin regeneration and blastema formation in the medaka, Oryzias latipes. Mech Dev 121, 861–872 (2004). 15210191 10.1016/j.mod.2004.03.015

[b12] Sun Y. *et al.* Mammalian target of rapamycin regulates miRNA-1 and follistatin in skeletal myogenesis. J Cell Biol 189, 1157–1169 (2010). 20566686 10.1083/jcb.200912093 PMC2894448

[b13] Zhang D. *et al.* Attenuation of p38-mediated miR-1/133 expression facilitates myoblast proliferation during the early stage of muscle regeneration. PLoS One 7, e41478 (2012). 22911796 10.1371/journal.pone.0041478 PMC3404058

[b14] Zou Y. *et al.* Developmental decline in neuronal regeneration by the progressive change of two intrinsic timers. Science 340, 372–376 (2013). 23599497 10.1126/science.1231321 PMC4074024

[b15] Oviedo N. J. & Beane W. S. Regeneration: The origin of cancer or a possible cure? Semin Cell Dev Biol 20, 557–564 (2009). 19427247 10.1016/j.semcdb.2009.04.005 PMC2706275

[b16] Huntley R. P. *et al.* The GOA database: gene Ontology annotation updates for 2015. Nucleic Acids Res 43, D1057–1063 (2015). 25378336 10.1093/nar/gku1113 PMC4383930

[b17] Jimeno-Yepes A. J., Sticco J. C., Mork J. G. & Aronson A. R. GeneRIF indexing: sentence selection based on machine learning. BMC Bioinformatics 14, 171 (2013). 23725347 10.1186/1471-2105-14-171 PMC3687823

[b18] Blanc V. *et al.* Apobec-1 complementation factor modulates liver regeneration by post-transcriptional regulation of interleukin-6 mRNA stability. J Biol Chem 285, 19184–19192 (2010). 20406809 10.1074/jbc.M110.115147 PMC2885197

[b19] Brown G. R. *et al.* Gene: a gene-centered information resource at NCBI. Nucleic Acids Res 43, D36–42 (2015). 25355515 10.1093/nar/gku1055 PMC4383897

[b20] Zhao M., Sun J. & Zhao Z. TSGene: a web resource for tumor suppressor genes. Nucleic Acids Res 41, D970–976 (2013). 23066107 10.1093/nar/gks937 PMC3531050

[b21] Kong L. *et al.* IQdb: an intelligence quotient score-associated gene resource for human intelligence. Database (Oxford) 2013, bat063 (2013). 10.1093/database/bat063 PMC3770929 24030781

[b22] Zhao M. *et al.* TSGene 2.0: an updated literature-based knowledgebase for tumor suppressor genes. Nucleic Acids Res 44, D1023–1031 (2016). 26590405 10.1093/nar/gkv1268 PMC4702895

[b23] Cerami E. G. *et al.* Pathway Commons, a web resource for biological pathway data. Nucleic Acids Res 39, D685–690 (2011). 21071392 10.1093/nar/gkq1039 PMC3013659

[b24] Jopling C. *et al.* Hypoxia induces myocardial regeneration in zebrafish. Circulation 126, 3017–3027 (2012). 23151342 10.1161/CIRCULATIONAHA.112.107888

[b25] Mimeault M. & Batra S. K. Concise review: recent advances on the significance of stem cells in tissue regeneration and cancer therapies. Stem Cells 24, 2319–2345 (2006). 16794264 10.1634/stemcells.2006-0066

[b26] Mimeault M., Mehta P. P., Hauke R. & Batra S. K. Functions of normal and malignant prostatic stem/progenitor cells in tissue regeneration and cancer progression and novel targeting therapies. Endocr Rev 29, 234–252 (2008). 18292464 10.1210/er.2007-0040 PMC2528844

[b27] Plikus M. V. *et al.* The Circadian Clock in Skin: Implications for Adult Stem Cells, Tissue Regeneration, Cancer, Aging, and Immunity. J Biol Rhythms 30, 163–182 (2015). 25589491 10.1177/0748730414563537 PMC4441597

[b28] Tataria M., Perryman S. V. & Sylvester K. G. Stem cells: tissue regeneration and cancer. Semin Pediatr Surg 15, 284–292 (2006). 17055959 10.1053/j.sempedsurg.2006.07.008

[b29] Natarajan A., Wagner B. & Sibilia M. The EGF receptor is required for efficient liver regeneration. Proc Natl Acad Sci USA 104, 17081–17086 (2007). 17940036 10.1073/pnas.0704126104 PMC2040457

[b30] Doan P. L. *et al.* Epidermal growth factor regulates hematopoietic regeneration after radiation injury. Nat Med 19, 295–304 (2013). 23377280 10.1038/nm.3070 PMC3594347

[b31] Normanno N. *et al.* Epidermal growth factor receptor (EGFR) signaling in cancer. Gene 366, 2–16 (2006). 16377102 10.1016/j.gene.2005.10.018

[b32] Jin Y. *et al.* The evolutionary dynamics of protein-protein interaction networks inferred from the reconstruction of ancient networks. PLoS One 8, e58134 (2013). 23526967 10.1371/journal.pone.0058134 PMC3603955

[b33] Zhao M., Chen L. & Qu H. CSGene: a literature-based database for cell senescence genes and its application to identify critical cell aging pathways and associated diseases. Cell Death Dis 7, e2053 (2016). 26775705 10.1038/cddis.2015.414 PMC4816187

[b34] Zhao M., Kong L., Liu Y. & Qu H. dbEMT: an epithelial-mesenchymal transition associated gene resource. Sci Rep 5, 11459 (2015). 26099468 10.1038/srep11459 PMC4477208

[b35] Zhao M. & Zhao Z. CNVannotator: a comprehensive annotation server for copy number variation in the human genome. PLoS One 8, e80170 (2013). 24244640 10.1371/journal.pone.0080170 PMC3828214

[b36] Zhao M., Ma L., Liu Y. & Qu H. Pedican: an online gene resource for pediatric cancers with literature evidence. Sci Rep 5, 11435 (2015). 26073932 10.1038/srep11435 PMC4466794

[b37] Zhao M., Liu Y. & O’Mara T. A. ECGene: A Literature-Based Knowledgebase of Endometrial Cancer Genes. Hum Mutat, 10.1002/humu.22950 (2015). PMC5066700 26699919

[b38] Liu Y., Xia J., Sun J. & Zhao M. OCGene: a database of experimentally verified ovarian cancer-related genes with precomputed regulation information. Cell Death Dis 6, e2036 (2015). 26720345 10.1038/cddis.2015.380 PMC4720911

[b39] Sayers E. W. *et al.* Database resources of the National Center for Biotechnology Information. Nucleic Acids Res 40, D13–25 (2012). 22140104 10.1093/nar/gkr1184 PMC3245031

[b40] Kozomara A. & Griffiths-Jones S. miRBase: integrating microRNA annotation and deep-sequencing data. Nucleic Acids Res 39, D152–157 (2011). 21037258 10.1093/nar/gkq1027 PMC3013655

[b41] Fernandez J. M., Hoffmann R. & Valencia A. iHOP web services. Nucleic Acids Res 35, W21–26 (2007). 17485473 10.1093/nar/gkm298 PMC1933131

[b42] Caspi R. *et al.* The MetaCyc database of metabolic pathways and enzymes and the BioCyc collection of Pathway/Genome Databases. Nucleic Acids Res 42, D459–471 (2014). 24225315 10.1093/nar/gkt1103 PMC3964957

[b43] Kanehisa M. *et al.* KEGG for linking genomes to life and the environment. Nucleic Acids Res 36, D480–484 (2008). 18077471 10.1093/nar/gkm882 PMC2238879

[b44] Schaefer C. F. *et al.* PID: the Pathway Interaction Database. Nucleic Acids Res 37, D674–679 (2009). 18832364 10.1093/nar/gkn653 PMC2686461

[b45] Zhao M. & Qu H. PathLocdb: a comprehensive database for the subcellular localization of metabolic pathways and its application to multiple localization analysis. BMC Genomics 11 Suppl 4, S13 (2010). 21143796 10.1186/1471-2164-11-S4-S13 PMC3005916

[b46] Thomas P. D. *et al.* PANTHER: a library of protein families and subfamilies indexed by function. Genome Res 13, 2129–2141 (2003). 12952881 10.1101/gr.772403 PMC403709

[b47] Croft D. *et al.* Reactome: a database of reactions, pathways and biological processes. Nucleic Acids Res 39, D691–697 (2011). 21067998 10.1093/nar/gkq1018 PMC3013646

[b48] Matthews L. *et al.* Reactome knowledgebase of human biological pathways and processes. Nucleic Acids Res 37, D619–622 (2009). 18981052 10.1093/nar/gkn863 PMC2686536

[b49] Kanehisa M. *et al.* KEGG for representation and analysis of molecular networks involving diseases and drugs. Nucleic Acids Res 38, D355–360 (2010). 19880382 10.1093/nar/gkp896 PMC2808910

[b50] Osborne J. D. *et al.* Annotating the human genome with Disease Ontology. BMC Genomics 10 Suppl 1, S6 (2009). 19594883 10.1186/1471-2164-10-S1-S6 PMC2709267

[b51] Du P. *et al.* From disease ontology to disease-ontology lite: statistical methods to adapt a general-purpose ontology for the test of gene-ontology associations. Bioinformatics 25, i63–68 (2009). 19478018 10.1093/bioinformatics/btp193 PMC2687947

[b52] Becker K. G. *et al.* The genetic association database. Nat Genet 36, 431–432 (2004). 15118671 10.1038/ng0504-431

[b53] Hindorff L. A. *et al.* Potential etiologic and functional implications of genome-wide association loci for human diseases and traits. Proc Natl Acad Sci USA 106, 9362–9367 (2009). 19474294 10.1073/pnas.0903103106 PMC2687147

[b54] Sayers E. W. *et al.* Database resources of the National Center for Biotechnology Information. Nucleic Acids Res 39, D38–51 (2011). 21097890 10.1093/nar/gkq1172 PMC3013733

[b55] Xie C. *et al.* KOBAS 2.0: a web server for annotation and identification of enriched pathways and diseases. Nucleic Acids Res 39, W316–322 (2011). 21715386 10.1093/nar/gkr483 PMC3125809

[b56] Wu J. *et al.* KOBAS server: a web-based platform for automated annotation and pathway identification. Nucleic Acids Res 34, W720–724 (2006). 16845106 10.1093/nar/gkl167 PMC1538915

[b57] Lee T. Y. *et al.* A comprehensive resource for integrating and displaying protein post-translational modifications. BMC Res Notes 2, 111 (2009). 19549291 10.1186/1756-0500-2-111 PMC2713254

[b58] Heinemeyer T. *et al.* Expanding the TRANSFAC database towards an expert system of regulatory molecular mechanisms. Nucleic Acids Res 27, 318–322 (1999). 9847216 10.1093/nar/27.1.318 PMC148171

[b59] Su A. I. *et al.* A gene atlas of the mouse and human protein-encoding transcriptomes. Proc Natl Acad Sci USA 101, 6062–6067 (2004). 15075390 10.1073/pnas.0400782101 PMC395923

[b60] Lv J. *et al.* DiseaseMeth: a human disease methylation database. Nucleic Acids Res 40, D1030–1035 (2012). 22135302 10.1093/nar/gkr1169 PMC3245164

[b61] Aerts S. *et al.* Gene prioritization through genomic data fusion. Nat Biotechnol 24, 537–544 (2006). 16680138 10.1038/nbt1203

[b62] Krzywinski M. *et al.* Circos: an information aesthetic for comparative genomics. Genome Res 19, 1639–1645 (2009). 19541911 10.1101/gr.092759.109 PMC2752132

[b63] Gao J. *et al.* Integrative analysis of complex cancer genomics and clinical profiles using the cBioPortal. Sci Signal 6, pl1 (2013). 23550210 10.1126/scisignal.2004088 PMC4160307

[b64] Zhao M., Li X. & Qu H. EDdb: a web resource for eating disorder and its application to identify an extended adipocytokine signaling pathway related to eating disorder. Sci China Life Sci 56, 1086–1096 (2013). 24302289 10.1007/s11427-013-4573-2

[b65] Barabasi A. L. & Oltvai Z. N. Network biology: understanding the cell’s functional organization. Nat Rev Genet 5, 101–113 (2004). 14735121 10.1038/nrg1272

[b66] Smoot M. E. *et al.* Cytoscape 2.8: new features for data integration and network visualization. Bioinformatics 27, 431–432 (2011). 21149340 10.1093/bioinformatics/btq675 PMC3031041

