# Supplementary material for: REGene: a literature-based knowledgebase of animal regeneration that bridge tissue regeneration and cancer
Source: Sci Rep. 2016 Mar 15;6:23167. doi: 10.1038/srep23167 (PMC4791596; doi:10.1038/srep23167)
Supplement: Supplementary Information [file srep23167-s1.pdf]

Supplementary figures:

REGene: a literature-based knowledgebase of animal regeneration that bridge tissue regeneration and cancer

Min Zhao, Bronwyn Rotgans, Tianfang Wang, S.F. Cummins[§]

School of Engineering, Faculty of Science, Health, Education and Engineering, University of the Sunshine Coast, Maroochydore DC, Queensland, Australia, 4558

[§]To whom correspondence should be addressed, Scott Cummins, scummins@usc.edu.au.

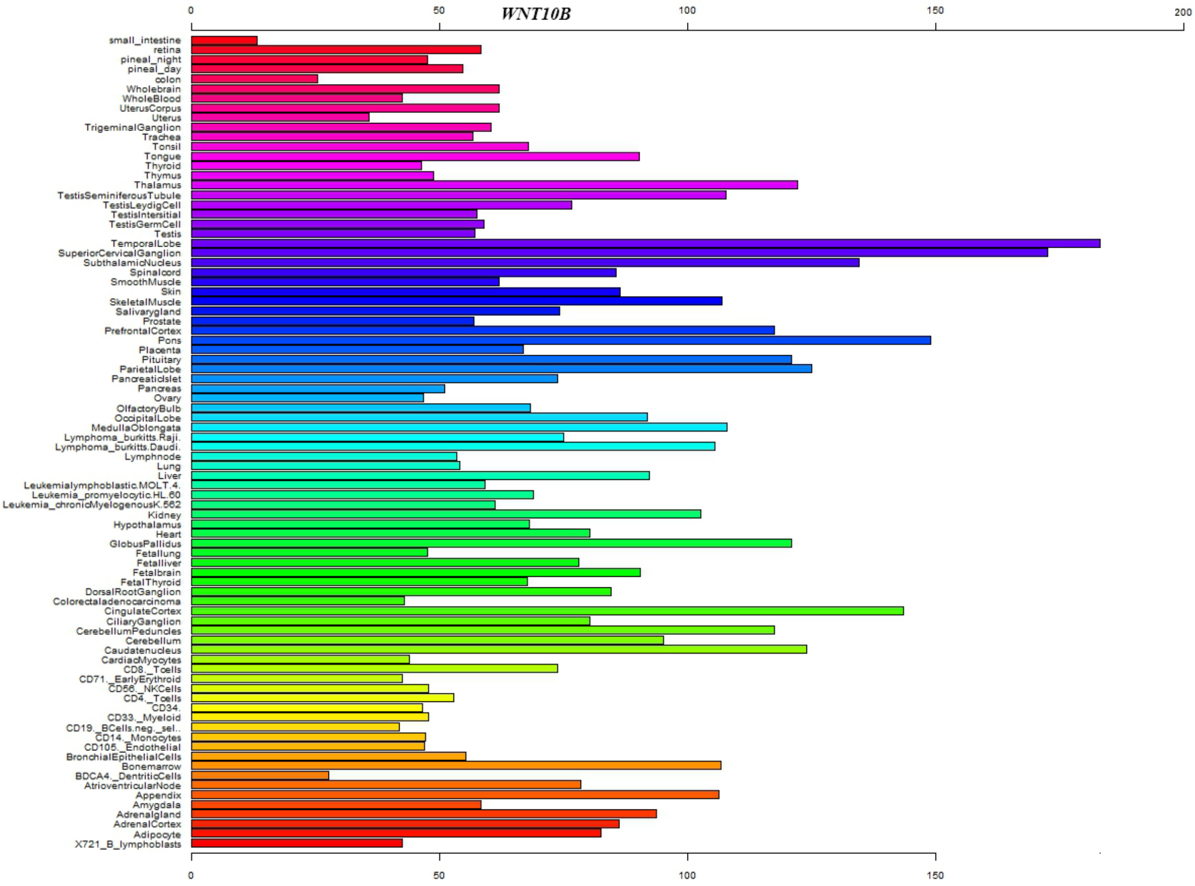

Figure S1 The gene expression profile on WNT10B.

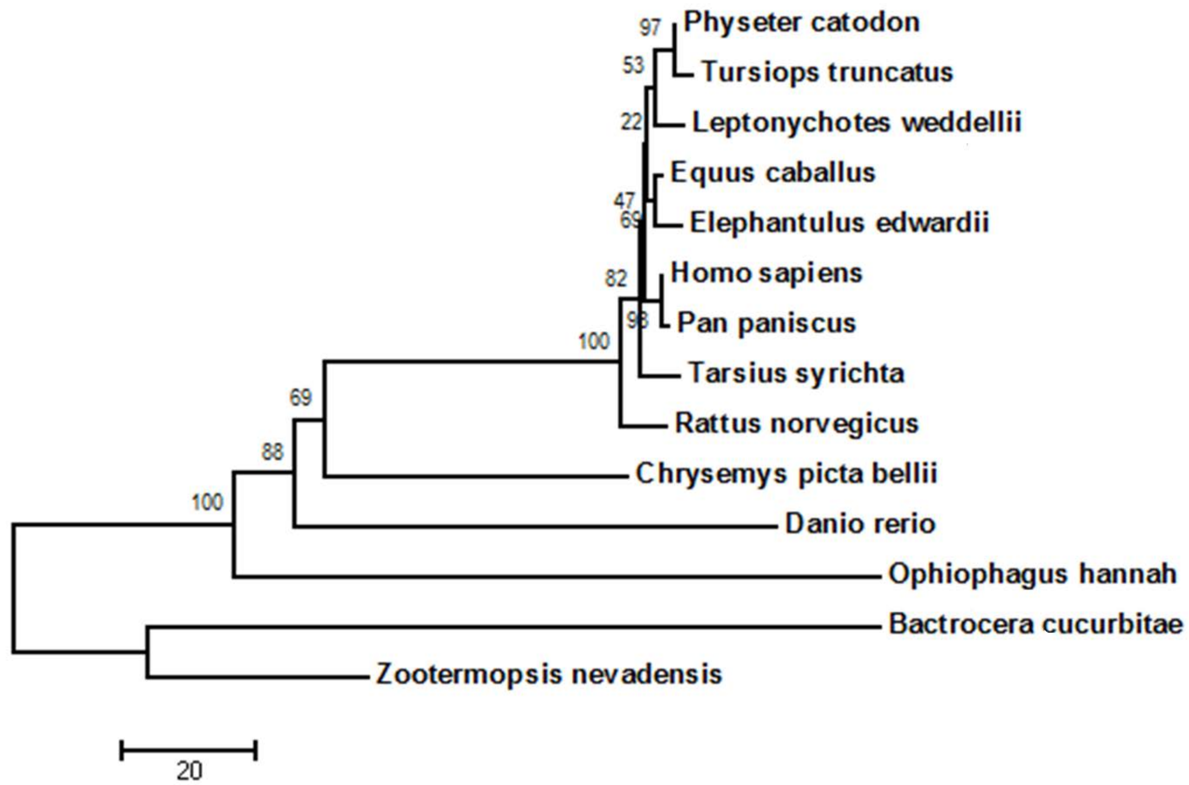

Figure S2 The phylogenetic tree analysis on WNT10B.

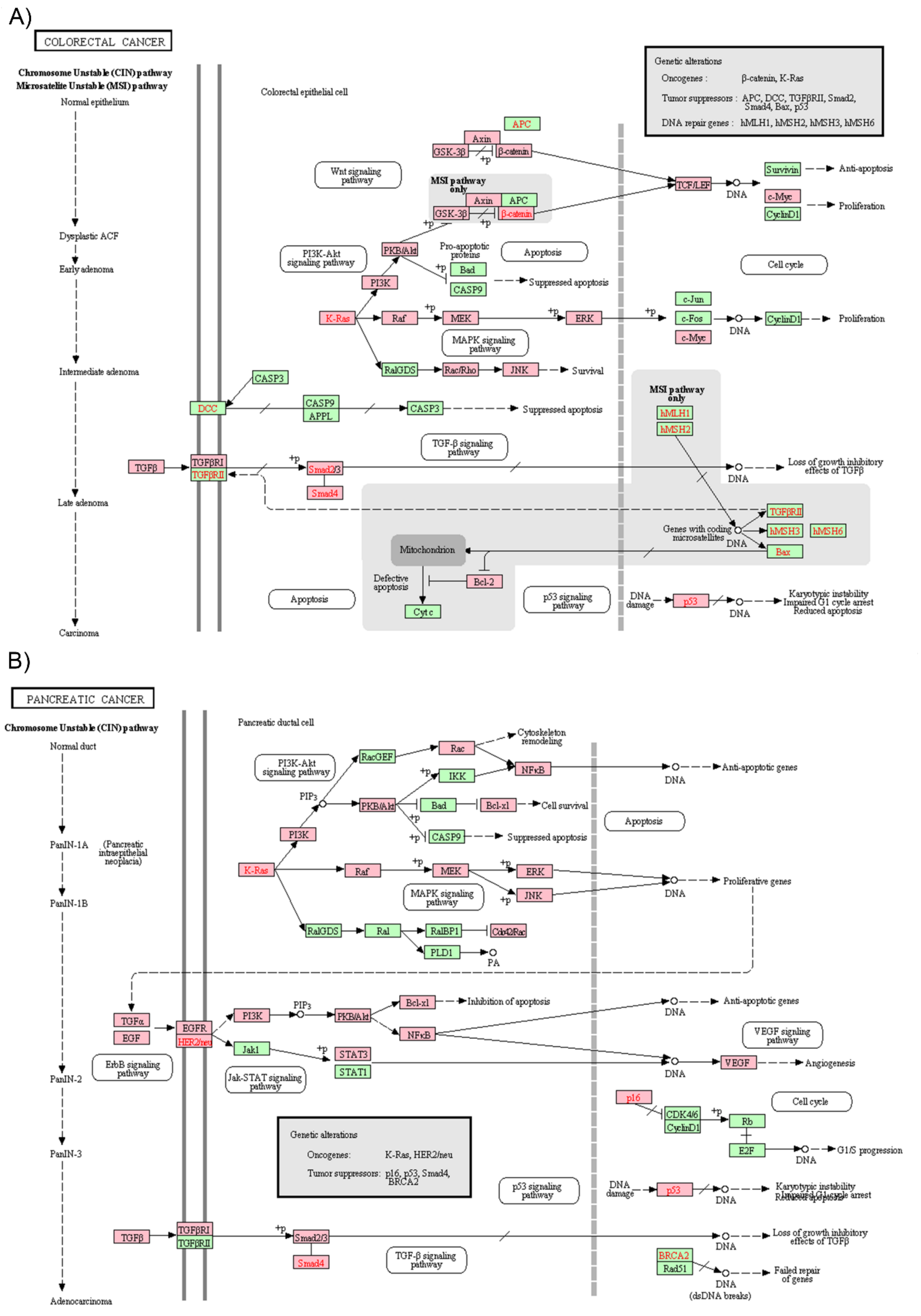

Figure S3 The regeneration-related genes in the KEGG colorectal and pancreatic cancer pathways.

Figure S4 The domain mutational pattern of the 19 reliable regeneration-related genes.

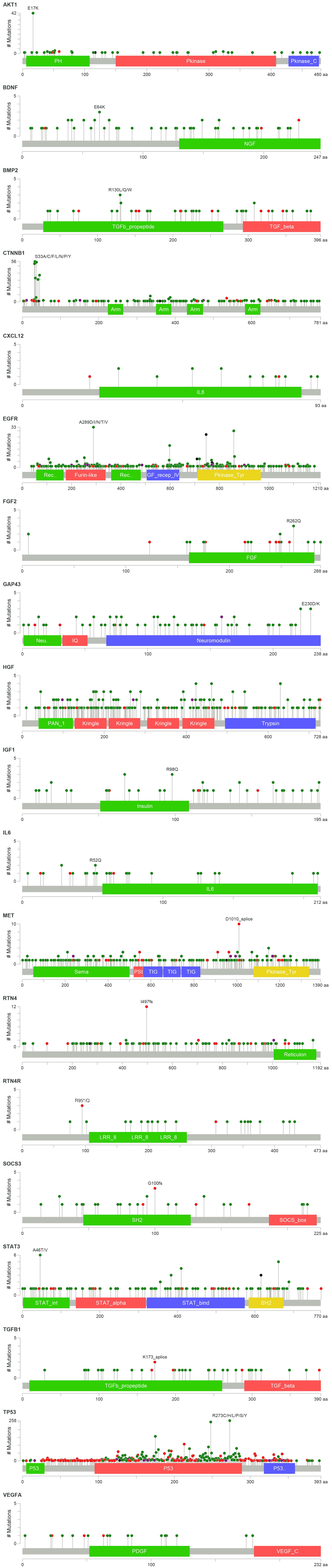
AKT1
E17K
Mutations
PH
Pkinase
Pkinase_C
480 aa
BDNF
E64K
NGF
247 aa
BMP2
R130L/Q/W
TGFb_propeptide
TGF_beta
396 aa
CTNNB1
S33A/C/F/L/N/P/Y
Arm
781 aa
CXCL12
IL8
93 aa
EGFR
A289D/I/N/T/V
Rec..
Furin-like
GF_recep_IV
Pkinase_Tyr
1210 aa
FGF2
R262Q
FGF
288 aa
GAP43
E230D/K
Neu..
IQ
Neuromodulin
238 aa
HGF
PAN_1
Kringle
Trypsin
728 aa
IGF1
R98Q
Insulin
195 aa
IL6
R52Q
IL6
212 aa
MET
D1010_splice
Sema
PSI
TIG
Pkinase_Tyr
1390 aa
RTN4
I497fs
Reticulon
1192 aa
RTN4R
R95*/Q
LRR_8
473 aa
SOCS3
G100fs
SH2
SOCS_box
225 aa
STAT3
A46T/V
STAT_int
STAT_alpha
STAT_bind
SH2
770 aa
TGFB1
K173_splice
TGFb_propeptide
TGF_beta
390 aa
TP53
R273C/H/L/P/S/Y
P53..
P53
393 aa
VEGFA
PDGF
VEGF_C
232 aa
